# R-spondin2 promotes hematopoietic differentiation of human pluripotent stem cells by activating TGF beta signaling

**DOI:** 10.1186/s13287-019-1242-9

**Published:** 2019-05-20

**Authors:** Yv Wang, Jie Gao, Hongtao Wang, Mengge Wang, Yuqi Wen, Jiaojiao Guo, Pei Su, Lihong Shi, Wen Zhou, Jiaxi Zhou

**Affiliations:** 1 State Key Laboratory of Experimental Hematology, Institute of Hematology and Blood Diseases Hospital, Tianjin, 300020 China; 2 Center for Stem Cell Medicine, Chinese Academy of Medical Sciences and Department of Stem Cells and Regenerative Medicine, Peking Union Medical College, Tianjin, 300020 China; 3 School of Basic Medical Science and Cancer Research Institute, Central South University, Changsha, 410013 China

**Keywords:** R-spondin2, Human pluripotent stem cells, Hematopoietic differentiation, Mesoderm, TGF-β signaling

## Abstract

**Background:**

Human pluripotent stem cells (hPSCs) provide supplies of potential functional blood cells to suffice the clinical needs. However, the underlying mechanism of generating genuine hematopoietic stem cells (HSCs) and functional blood cells from hPSCs remains largely elusive.

**Method:**

In this study, we supplied R-spondin2 exogenously during hematopoietic differentiation of hPSCs under various culture conditions and analyzed the production of hematopoietic progenitor cells (HPCs). We further added R-spondin2 at different temporal window to pin down the stage at which R-spondin2 conferred its effects. RNA-SEQ-based gene profiling was applied to analyze genes with significantly altered expression and altered signaling pathways. Finally, megakaryocytic differentiation and platelet generation were determined using HPCs with R-spondin2 treatment.

**Results:**

We found that R-spondin2 generated by hematopoiesis-supporting stromal cells significantly enhances hematopoietic differentiation of hPSCs. Supply of R-spondin2 exogenously at the early stage of mesoderm differentiation elevates the generation of APLNR^+^ cells. Furthermore, early treatment of cells with R-spondin2 enables us to increase the output of hPSC-derived platelet-like particles (PLPs) with intact function. At the mechanistic level, R-spondin2 activates TGF-β signaling to promote the hematopoietic differentiation.

**Conclusions:**

Our results demonstrate that a transient supply of R-spondin2 can efficiently promote hematopoietic development by activating both WNT and TGF-β signaling. R-spondin2 can be therefore used as a powerful tool for large-scale generation of functional hematopoietic progenitors and platelets for translational medicine.

**Electronic supplementary material:**

The online version of this article (10.1186/s13287-019-1242-9) contains supplementary material, which is available to authorized users.

## Background

Hematopoietic stem cell transplantation and blood cell transfusion such as the use of red blood cells and platelet have been widely applied as potent treatments of multiple blood disorders [1]. The limited availability of suitable HLA-matched donors and blood donors, especially in China, the modest expansion capability of LT-HSCs after chemotherapy treatment during allogenic transplantation, incidences of graft failure and GVHD (graft-versus-host disease) in haploidentical HSC transplantation, a short shelf life of platelets from blood donors, and potential infections or associated complications are currently limiting the application of these strategies, calling for a new and abundant source for blood cells [1–5]. Human pluripotent stem cells (hPSCs), including human embryonic stem cells (hESCs) and induced pluripotent stem cells (iPSCs), serve as a potential source of various blood cells due to their capability of long-term self-renewal and multipotent differentiation [6–9]. This feature provides a promising alternative to the production of hematopoietic cells, such as hematopoietic stem cells (HSCs), platelets, and red blood cells. However, despite of numerous attempts to recapture the hematopoietic developmental process in vivo, the generation of functional blood cells remains at a low level of efficacy and quality [2], thereby calling for a more thorough understanding of the regulatory mechanism of hematopoietic differentiation and the development of more robust strategies for blood cell generation.

During embryonic development in vivo, hematopoietic development occurs from the mesoderm, which gives rise to both hematopoietic and vascular lineages [10]. Hemogenic endothelium (HE) has been identified as the direct precursor of HSCs following an endothelium-hematopoietic transition (EHT) [11]. hPSC hematopoietic differentiation in vitro goes through similar stages, mimicking the in vivo progress of hematopoietic development. Multiple studies have shown that every stage of hematopoietic differentiation is under precise regulation by several key signaling pathways [5, 8, 10]. For example, BMP4 is crucial for induction of primitive streak from hESCs [12, 13], while formation of mesodermal cells also depends on synergistic regulation of the canonical WNT/β and Activin/Nodal signaling pathways [14, 15]. VEGF is necessary and sufficient for specifying HE cells, and bFGF acts synergistically with VEGF [16]. In addition, the NOTCH signaling pathway plays a vital role in specifying arterial-type definitive HE from hPSCs [17]. In the subsequent process of EHT, inhibition of the TGFβ signaling pathway is required for generating hematopoietic progenitors [16, 18]. Because of the pivotal roles of these signaling pathways, identification of novel components of the pathways and manipulation of the factors should benefit the efficiency of hematopoietic differentiation.

In addition to the intracellular signaling pathways, accumulating evidence has revealed the significance of extracellular factors in hematopoietic differentiation. During embryonic development and in adulthood, a wide range of soluble factors secreted from various cells exerts influences on development and maintenance of HSCs [19–22]. Recent studies also emphasized the importance of extracellular paracrine factors in hematopoietic development in vitro. Menendez et al. reported that mesenchymal stem cell (MSC)-conditioned media augments hematopoietic specification from hESCs, implying an important role of secreted microenvironmental factors [23]. Tenascin C, an extracellular matrix protein identified from OP9 feeder expression profiling, has been used in an hPSC hematopoietic differentiation system to enhance HEP generation and definitive hematopoiesis [24]. Extracellular CXCL12/CXCR4 signaling can further confer hPSC-derived hematopoietic progenitor cells function of in vivo transplantation [25]. To date, the mechanisms by which these extracellular factors function have not been well defined. Thus, exploring novel extracellular factors and unraveling their connection to key intracellular pathways should prove helpful in revealing the mechanisms of hematopoietic development and ameliorate the strategy of blood cell production in vitro.

We recently conducted RNA-SEQ screening and successfully identified new factors in controlling hPSC hematopoietic differentiation, including MEIS1 (Myeloid Ectopic Viral Integration Site 1 homolog) and MEIS2 (Myeloid Ectopic Viral Integration Site 2 homolog), which regulate HEP generation and EHT, respectively [26, 27]. In the current study, we have further revealed R-spondin2 as a key modulator of early hematopoietic differentiation of hPSCs, which augments APLNR^+^ mesoderm cell generation. We have also identified TGFβ signaling as a novel downstream target of R-spondin2, which works in parallel to WNT signaling to mediate the effects of R-spondin2 on hPSC hematopoietic differentiation.

## Material and methods

### hPSC cultivation

H1 hESC line (WiCell Research Institute, Madison, WI), H9 hESC line (WiCell Research Institute, Madison, WI), BC1 hiPSCs (from Dr. Linzhao Cheng) [28], and Z-15 hiPSCs (from Dr. Zhijian Xiao) [29–31] were used in this study. BC1 cells were derived from BM CD34^+^ cells reprogrammed by OCT4, SOX2, KLF4, c-MYC, and LIN28 and characterized with pluripotency markers, karyotyping, in vitro pluripotency assay of embyroid body formation, and in vivo pluripotency assay of teratoma formation [28]. Z-15 cells were derived from peripheral blood mononuclear cells reprogrammed by OCT4, SOX2, KLF4, c-MYC, and BCL-XL and characterized with pluripotency markers, karyotyping, and in vivo pluripotency assay of teratoma formation [30, 31]. hPSCs were cultured on Matrigel-coated plates (Corning) in mTeSR1 (Stem cell Technology) to maintain a pluripotent state. Medium was changed daily, and colonies were passaged every 4 days with 2 U/mL Dispase (Sigma) according to the manufacturer’s instructions.

### Hematopoietic differentiation from hPSCs in mAGM-S3 co-culture

Hematopoietic differentiation from hPSCs in mAGM-S3 co-culture system was performed as described earlier [26]. hPSCs were first dissociated into single cells with Accutase (Sigma) and seeded at the density of 6 × 10^4^/mL with 10 μM ROCK inhibitor Y-27632. After 48 h of culture, when cells grew into small colonies with similar sizes, the hPSC colonies were passaged and plated onto over-confluent mAGM-S3 feeder cells. Forty-eight hours later, medium was replaced with IMDM hematopoietic differentiation medium containing 10% fetal bovine serum. Media were changed every day for up to 14 days. Re-constituted R-spondin2 was obtained from Peprotech. R-spondin2 was added directly to the culture medium at 20 ng/mL at day 0 of hematopoietic differentiation.

### Hematopoietic differentiation from hPSCs in chemical defined system

Hematopoietic differentiation from hPSCs in chemically defined system was performed as described before [26]. hPSCs were dissociated into single cells with Accutase and seeded with Y-27632 onto 12-well plates coated with growth factor-reduced Matrigel (Corning) at the density of 3.5 × 10^4^ cells/mL. After 24 h, hematopoietic differentiation was performed using a stepwise induction procedure. Medium was replaced with Custom mTeSR1 (Stem cell Technology) supplemented with BMP4 (5 ng/mL) and Activin A (5 ng/mL) for days 0–2, VEGF (40 ng/mL) and bFGF (50 ng/mL) for days 2–4, and VEGF, bFGF, and SB431542 (20uM)(STEMGENT) for days 4–7. All cytokines used were from PeproTech. R-spondin2 was added directly to the culture medium at 20 ng/mL at day 0 of hematopoietic differentiation.

### Flow cytometry

To determine the proportion of defined populations in aforementioned differentiation culture conditions, differentiated cells were disassociated with 0.25% trypsin-EDTA, and cell suspension was stained with fluorescein-conjugated antibodies as follows: TRA-1-85-APC, hAPLNR-APC, hCD31-PE, hCD34-APC, hCD43-APC, and hCD45-PE. Detailed information for the antibodies is listed in Additional file 1: Table S2. Before analysis, cells were filtered through 70 μm cell strainer to obtain single cell suspension and stained with DAPI to exclude dead cells. Flow cytometry analysis was performed using a FACS CantoII flow cytometer, and cell sorting was performed using FACS Aria III sorter (BD Biosciences).

### Immunofluorescence

Cultured cells were fixed with 4% PFA in PBS for 20 min, permeabilized with 0.1% Triton X-100 in PBS for 20 min, and blocked with 1–5% BSA in PBS for 1 h. Cells were then incubated with hCD43 and hCD45 primary antibodies at 4 °C overnight. After washes of PBS, cells were incubated with fluorescent anti-mouse secondary antibody conjugated with Alexa Fluor 488 or 594 at room temperature (RT) for 1 h. The nuclei were stained with DAPI for 10 min before experimentation. The cells were examined and recorded using a fluorescence microscope. Information and dilution for the antibodies are listed in Additional file 1: Table S1.

### Colony-forming unit (CFU) assay

CFU assays were performed by plating 5 × 10^3^ cells/well from single cells obtained from day 12 of co-culture into methylcellulose H4435 in a 24-well plate. Cells were incubated at 37 °C for 14 days before the colonies were counted based on standard morphological criteria. BFU-E (burst-forming unit-erythroid), CFU-E (colony-forming unit-erythrocyte), CFU-GM (colony-forming unit-granulocyte/macrophage), and CFU-GEMM (colony-forming unit-granulocyte/erythroid/macrophage/monocyte) were classified and enumerated based on morphological recognition.

### Western blotting

Western blotting analysis was performed as described previously [26]. hPSCs were lysed directly on ice in Laemmli sample buffer (BioRad) supplemented with phosphatase inhibitor cocktail. The cell lysates were electrophoresed on 10% SDS-PAGE gel and transferred onto PVEF membranes. The membranes were blocked at RT with 5% nonfat milk in 0.1% Tween-20 in PBS (PBST) for 1 h and then incubated with primary antibodies overnight at 4 °C. After being washed with TBST, membranes were then incubated with HRP-coupled secondary antibodies for 2 h at room temperature and detected using the Super-Signal West Pico Chemiluminescent Substrate (Thermo) in ImageQuant LAS-4010 (GE). GAPDH was used as a loading control. Dilutions for various antibodies are shown in Additional file 1: Table S1. Densitometric quantitation of Western blotting results was performed with ImageJ software. Western blotting images were quantified by analyzing the intensities of greyscales of each band.

### Quantitative real-time PCR

RNA was extracted with the Trizol according to the manufacturer’s instructions. RNA was transcribed into cDNA using random primers. Real-time PCR was carried out with SYBR green detection on an ABI 7900HT Fast Real-Time PCR cycler. Relative quantification of transcript levels was calculated using CT values normalized to ACTIN. Sequences for various primer pairs are shown in Additional file 1: Table S3.

### RNA-SEQ

RNA-SEQ analysis was performed as previously described [26]. All human cells sorted with hTRA-1-85^+^ from day 2 of co-culture were enriched and analyzed by RNA-SEQ. The expression levels were visualized using a heatmap built based on the value of log10 (FPKM+1). GO enrichment and GSE analyses were performed. The data are available at Gene Expression Omnibus (GEO) (Accession number GEO: GSE118596).

### Statistical analysis

At least three independent experiments were performed for each analysis. The number of biological replicates is indicated by the *n* value. All graphs depict mean ± SD. Statistical analysis was performed using a two-tailed unpaired Student’s *t* test, and the results were considered statistically significant at *P* value < 0.05 and were denoted as NS, not significant; *P < 0.05; ** *P* < 0.01; ***P < 0.001.

The graphs and statistical evaluation were performed using GraphPad Prism (GraphPad Software).

## Results

### R-spondin2 promotes generation of hematopoietic progenitors from hESCs

**Fig. 1:**
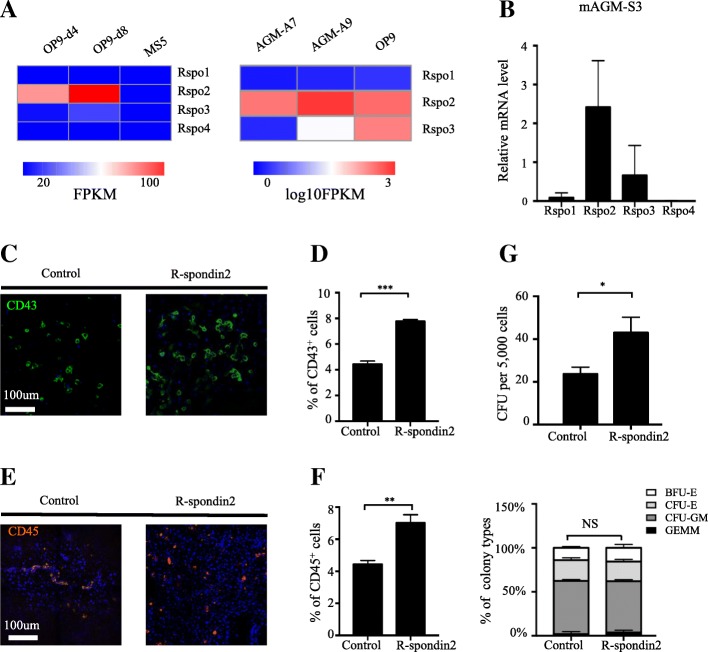
R-spondin2 promotes generation of hematopoietic progenitors from hESCs. **a** Left panel: heatmap of Rspo expression in OP9-d4, OP9-d8, and MS5 stromal cells (accession number GEO: GSE61580). Right panel: heatmap of Rspo expression in AGM-S3-A7, AGM-S3-A9 subclones of AGM-S3 stromal cell and OP9 cells (accession number GEO: GSE11891). **b** Real-time PCR analysis of expression of Rspos in mAGM-S3 stromal cells. Relative expression is normalized to the level (= 1) of Actin. Results are shown as means ± SD (*n* = 3). **c** Representative immunofluorescence images of H1 cells with or without treatment of R-spondin2 (20 ng/mL) showing the generation of CD43^+^ HPCs at day 7 of mAGM-S3 co-culture. **d** Flow cytometry analysis of H1 cells with or without treatment of R-spondin2 (20 ng/mL) showing the generation of CD43^+^ HPCs at day 7 of mAGM-S3 co-culture. Results are shown as means ± SD (*n* = 3). ***P < 0.001. **e** Representative immunofluorescence images of H1 cells with or without the treatment of R-spondin2 (20 ng/mL) showing the generation of CD45^+^ HPCs at day 10 of mAGM-S3 co-culture. **f** Flow cytometry analysis of H1 cells with or without the treatment of R-spondin2 (20 ng/mL) showing the generation of CD45^+^ HPCs at day 10 of mAGM-S3 co-culture. Results are shown as means ± SD (*n* = 3). **P < 0.01. **g** Hematopoietic colony-forming potential of hESC-derived cells after 12 days of co-culture with or without the treatment of R-spondin2 (20 ng/mL), analyzed using total colony numbers (upper panel) and the proportion of BFU-E (burst-forming unit-erythroid), CFU-E (colony forming unit-erythrocyte), CFU-GM (colony forming unit-granulocyte/macrophage) and CFU-GEMM (colony-forming unit-granulocyte/erythroid/macrophage/monocyte) (lower panel). Results are shown as means ± SD (*n* = 3). NS, not significant, *P < 0.05. GEMM, *P* = 0.56; GM, *P* = 0.32; CFU-E, *P* = 0.65; BFU-E, *P* = 0.64

To discover novel regulators of hPSC early hematopoietic differentiation, we recently conducted RNA-SEQ screening and identified the role of MEIS1 and MEIS2 in modulating formation of HEP from mesoderm cells and in EHT, respectively [26, 27]. In the current study, we focused on the identification of potential extracellular regulators. We initially speculated that cytokines or growth factors may be produced by hematopoietic differentiation supporting stromal cells including mAGM-S3 and OP9—two cell lines extensively used for hematopoietic differentiation of hPSCs in a variety of studies including ours [10, 26]. Interestingly, from the published RNA-seq results [24, 32], we discovered high expression of members of R-spondin family that are well-known WNT signaling agonists (Fig. 1a) [33–36]. R-spondin family includes four members: R-spondin1 to R-spondin4 [33, 37]. Their expression was measured in mAGM-S3 cells, enabling us to find that R-spondin2 exhibited the highest expression among four members (Fig. 1b). Thus, we chose R-spondin2 for further functional studies.

To test the potential role of R-spondin2 in hESC hematopoietic differentiation, we added human R-spondin2 (20 ng/mL) to hESCs induced to undergo hematopoietic differentiation by mAGM-S3 co-culture. Indeed, we found that the addition of R-spondin2 significantly enhanced the appearance of cobble-stone-like hematopoietic progenitors from H1 hESCs (Additional file 2: Figure S1A). Immunofluorescence and flow cytometry analyses showed that much more CD43^+^ HPCs were generated upon R-spondin2 treatment in H1 cells (Additional file 2: Figure S1B, Fig. 1c, d; control 4.50% ± 0.12% vs R-spondin2 7.78% ± 0.07%, *P* < 0.001). This stimulatory effect was dose-dependent (Additional file 2: Figure S1C). In addition, a greater number of CD45^+^ HPCs were derived upon R-spondin2 treatment (Fig. 1e, f; control 4.44% ± 0.14% vs R-spondin2 7.03% ± 0.29%, *P* < 0.01; Additional file 2: Figure S1D, S1E). These results suggest that R-spondin2 is capable of enhancing hematopoietic differentiation of hESCs in a dosage-dependent manner. To further assess the differentiation potential of generated hematopoietic progenitors, we utilized the colony-forming unit (CFU) assay and found significantly elevated number of total colonies after R-spondin2 treatment (Fig. 1g, upper panel; control 23.7 ± 1.9 vs R-spondin2 43 ± 4.2, *P* < 0.05). However, there was minimal change in the percentage of various types of colonies (Fig. 1g, lower panel, Additional file 2: Figure S1F). Thus, R-spondin2 enhances the generation of hematopoietic progenitors from hPSCs without changing the colony-forming capacity.

### R-spondin2 enhances hematopoietic differentiation of hPSCs independently of culture conditions and cell lines

**Fig. 2:**
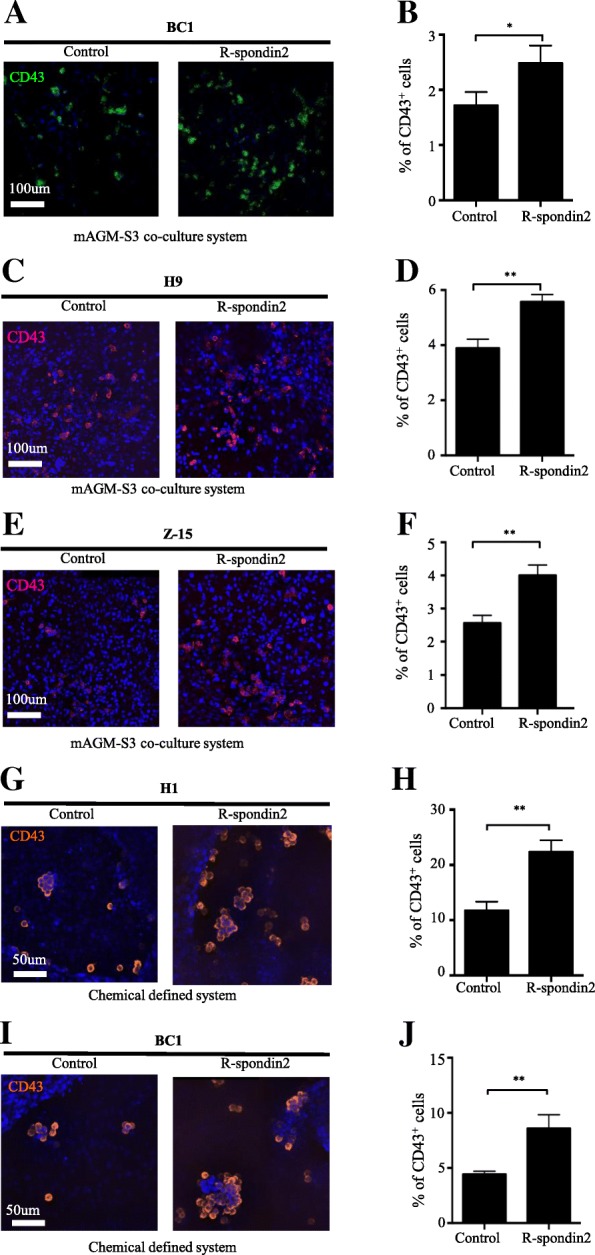
R-spondin2 enhances hematopoietic differentiation of hPSCs independently of culture conditions and cell lines. **a** Representative immunofluorescence images of BC1 cells with or without treatment of R-spondin2 (20 ng/mL) showing the generation of CD43^+^ HPCs at day 7 of mAGM-S3 co-culture. **b** Flow cytometry analysis of BC1 cells with or without treatment of R-spondin2 (20 ng/mL) showing the generation of CD43^+^ HPCs at day7 of mAGM-S3 co-culture. Results are shown as means ± SD (*n* = 3). *P < 0.05. **c** Representative immunofluorescence images of H9 cells with or without treatment of R-spondin2 (20 ng/mL) showing the generation of CD43^+^ HPCs at day7 of mAGM-S3 co-culture. **d** Flow cytometry analysis of H9 cells with or without treatment of R-spondin2 (20 ng/mL) showing the generation of CD43^+^ HPCs at day7 of mAGM-S3 co-culture. Results are shown as means ± SD (*n* = 3). **P < 0.01. **e** Representative immunofluorescence images of Z-15 cells with or without treatment of R-spondin2 (20 ng/mL) showing the generation of CD43^+^ HPCs at day7 of mAGM-S3 co-culture. **f** Flow cytometry analysis of Z-15 cells with or without treatment of R-spondin2 (20 ng/mL) showing the generation of CD43^+^ HPCs at day7 of mAGM-S3 co-culture. Results are shown as means ± SD (*n* = 3). **P < 0.01. **g** Representative immunofluorescence images of H1 cells with or without treatment of R-spondin2 (20 ng/mL) showing the generation of CD43^+^ HPCs at day 7 of chemical defined hematopoietic differentiation. **h** Flow cytometry analysis of H1 cells with or without treatment of R-spondin2 (20 ng/mL) showing the generation of CD43^+^ HPCs at day7 of chemical defined hematopoietic differentiation. Results are shown as means ± SD (*n* = 3). **P < 0.01. **i** Representative immunofluorescence images of BC1 cells with or without treatment of R-spondin2 (20 ng/mL) showing the generation of CD43^+^ HPCs at day 7 of chemically defined hematopoietic differentiation condition. **j** Flow cytometry analysis of BC1 cells with or without treatment of R-spondin2 (20 ng/mL) showing the generation of CD43^+^ HPCs at day 7 of chemically defined hematopoietic differentiation condition. Results are shown as means ± SD (*n* = 3). **P < 0.01

To exclude the possibility that the elevated hematopoietic differentiation conferred by R-spondin2 is cell line specific, we measured the effect of R-spondin2 on a human iPSC line BC1. Similar to the results from H1 cells, immunofluorescence and flow cytometry analyses also showed that much more CD43^+^ HPCs were generated with R-spondin2 treatment of BC1 cells (Fig. 2a, b; control 1.72% ± 0.10% vs R-spondin2 2.48% ± 0.16%, *P* < 0.05). In addition, the proportion of CD45^+^ HPCs was elevated upon R-spondin2 treatment in BC1 cells (Additional file 3: Figure S2A-S2B; control 1.67% ± 0.38% vs R-spondin2 3.27% ± 0.46%, *P* < 0.05). Therefore, R-spondin2 also promotes hematopoietic differentiation from BC1 cells. In addition, we tested R-spondin2 function in two more hPS cell lines, H9 hES and Z-15 hiPS cells. In line with our observations for H1 and BC1 cells, R-spondin2 treatment enhanced the generation of CD43^+^ HPCs from both H9 and Z-15 cells (Fig. 2c–f; H9 control 9.73 ± 0.47% vs R-spondin2 13.93 ± 0.38%, *P* < 0.01; Z-15 control 5.12 ± 0.27% vs R-spondin2 8.00 ± 0.36%, *P* < 0.01). These results strongly suggest that R-spondin2 promotes hematopoietic differentiation independently of cell lines.

Furthermore, to exclude the possibility that the elevated differentiation solely depended upon the mAGM-S3 culture system, we tested the effect of R-spondin2 in a chemically defined system described previously by us [26]. Consistently, a similar increase of CD43^+^ HPCs was also observed for H1 cells with R-spondin2 treatment under this condition (Fig. 2g, h; control 11.77% ± 0.92% vs R-spondin2 22.33% ± 1.24%, *P* < 0.01) and BC1 cells (Fig. 2i, j; control 4.42% ± 0.16% vs R-spondin2 8.58% ± 0.72%, *P* < 0.01). In addition, we also verified the results in H9 and Z-15 cells (Additional file 3: Figure S2C-F; H9 control 4.57 ± 0.52% vs R-spondin2 7.59 ± 0.36%, *P* < 0.01; Z-15 control 7.44 ± 0.22% vs R-spondin2 11.4 ± 0.44%, *P* < 0.01). Besides, we analyzed the number of CD43^+^ HPCs generated per hPSC from all the above four cell lines in both culture systems and observed a similar increase of CD43^+^ HPC number generated from each hPSC in both differentiation system (Additional file 3: Figure S2G-H). By using four hPS cell lines including H1 and H9 hESCs, BC1, and Z-15 hiPSCs, we showed that R-spondin2 promotes hematopoietic differentiation of these cells in both the co-culture and chemical defined differentiation systems.

### R-spondin2 treatment during early mesoderm differentiation suffices to promote hPSC hematopoietic differentiation

**Fig. 3:**
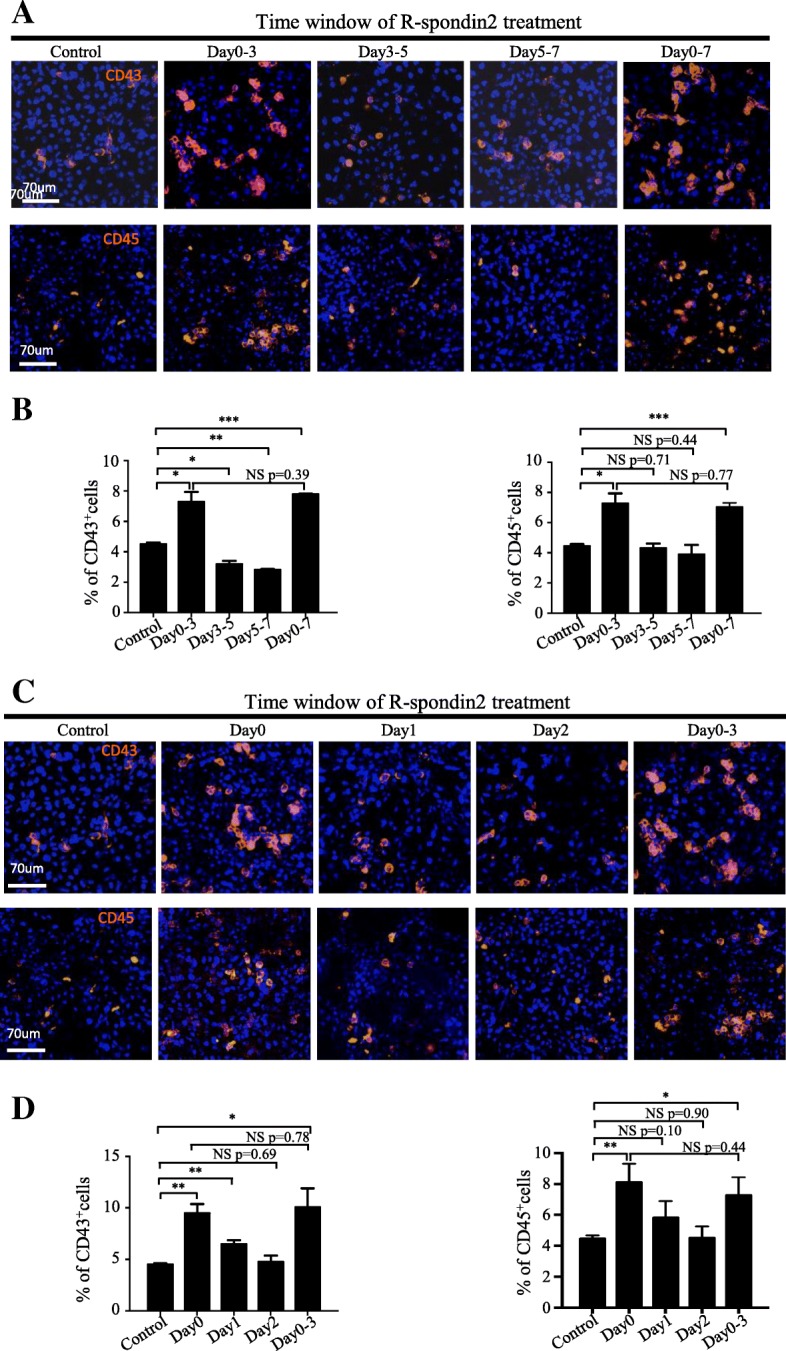
R-spondin2 treatment during early mesoderm differentiation suffices to promote hPSC hematopoietic differentiation. **a** Representative immunofluorescence images of H1 cells with treatment of R-spondin2 (20 ng/mL) at different stages of differentiation showing the generation of CD43^+^ HPCs at day 7 (top) and CD45^+^ HPCs at day 10 (bottom) of mAGM-S3 co-culture differentiation. **b** Flow cytometry analysis of the percentage of CD43^+^ HPCs at day 7 (left) and CD45^+^ HPCs at day 10 (right) from H1 cells in mAGM-S3 co-culture differentiation with R-spondin2 treatment (20 ng/mL) at different stages of differentiation. Day0–3: R-spondin2 was added from day 0 to day 3 and withdrawn at other days. Day3–5: R-spondin2 was added from day 3 to day 5 and withdrawn at other days. Day5–7: R-spondin2 was added from day 5 to day 7 and withdrawn at other days. Day0–7: R-spondin2 was added from day 0 to day 7 throughout. Results are shown as means ± D (*n* = 3). NS, not significant, *P < 0.05, ***P < 0.001. **c** Representative immunofluorescence images of H1 cells with the treatment of R-spondin2 (20 ng/mL) at different temporal window of mesoderm induction showing the generation of CD43^+^ HPCs at day 7 (top) and CD45^+^ HPCs at day 10 (bottom) of mAGM-S3 co-culture differentiation. **d** Flow cytometry analysis of the percentage of CD43^+^ HPCs at day 7 (left) and CD45^+^ HPCs at day 10 (right) from H1 cells in mAGM-S3 co-culture differentiation with R-spondin2 treatment (20 ng/mL) at different temporal window of mesoderm induction. Day0: R-spondin2 was added at day 0 for 24 h and withdrawn at other days. Day1: R-spondin2 was added at day 1 for 24 h and withdrawn at other times. Day2: R-spondin2 was added at day 2 for 24 h and withdrawn at other times. Day0–3: R-spondin2 was added from day 0 to day 3 and withdrawn at other times. Results are shown as means ± SD (*n* = 3). NS, not significant, *P < 0.05, **P < 0.01

As described in our previous studies, hematopoietic differentiation from hPSCs goes through a stepwise process including three stages—mesoderm induction (days 0–3), HE progenitor emergence (days 3–5), and HPC generation (days 5–7) [26]. Accordingly, we added R-spondin2 at these different stages to identify the stage(s) at which R-spondin2 exerts its functions (Additional file 4: Figure S3A). Interestingly, R-spondin-2 addition during days 0–3 led to the generation of the highest percentage of CD43^+^ and CD45^+^ HPCs compared to the treatment during other stages, as revealed by immunofluorescence studies (Fig. 3a). Little enhancement of differentiation was observed when R-spondin2 was added during the other two stages. Quantification with flow cytometry analysis further confirmed the observations (Fig. 3b). Similar results were also observed in BC1 cells (Additional file 4: Figure S3B). To further narrow down the temporal window of R-spondin2 function, we included R-spondin2 at different time points of mesoderm for duration of 1 day (Additional file 4: Figure S3C). Surprisingly, we found that the addition of R-spondin2 at the first day of differentiation was capable of significantly enhancing CD43^+^ HPC generation in both H1 and BC1 cells. The effect was comparable to that of consecutive R-spondin2 treatment from day 0 to day 3 (Fig. 3c, d, Additional file 4: Figure S3D). In contrast, the addition of R-spondin2 at the second day or third day after induction of differentiation only induced minor or no enhancement of CD43^+^ HPC generation (Fig. 3c, d, Additional file 4: Figure S3D). Together, our results showed that transient treatment of R-spondin2 at the early stage of mesoderm induction is sufficient to achieve maximal enhancement of hPSC hematopoietic differentiation.

### R-spondin2 augments APLNR^+^ mesodermal cells

**Fig. 4:**
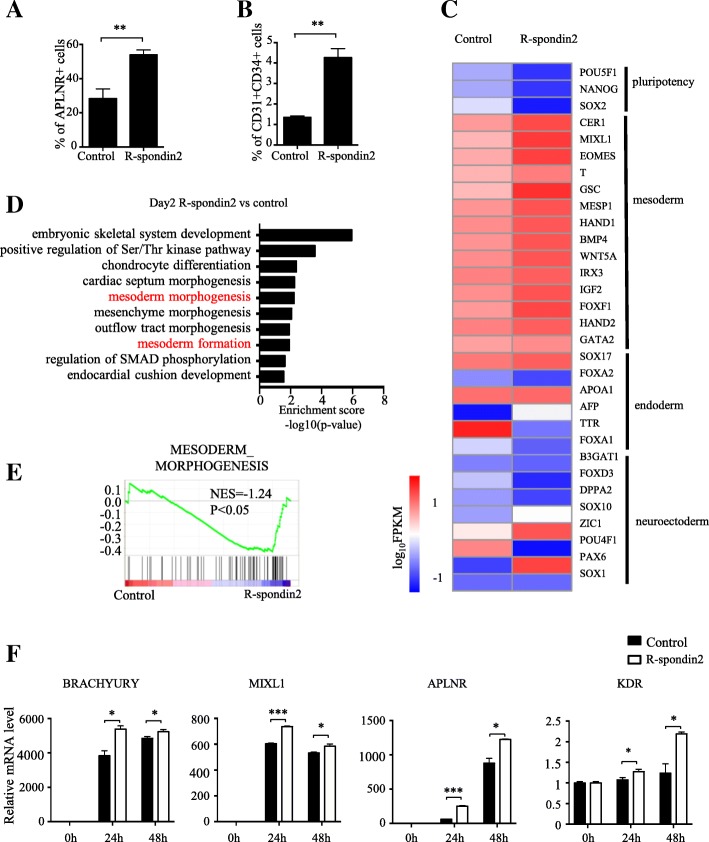
R-spondin2 promotes hematopoietic differentiation by augmenting APLNR+ mesodermal cells. **a**, **b** Flow cytometry analysis of the percentage of APLNR^+^ mesoderm cells (**a**) at day 3 and CD31^+^CD34^+^ HEPs (**b**) at day 5 of differentiation in mAGM-S3 co-culture from H1 cells with or without the treatment of R-spondin2 (20 ng/mL). Results are shown as means ± SD (*n* = 3). **P < 0.01. **c** Heatmap showing expression change of genes associated with pluripotency and three germ layers after R-spondin2 treatment (log_10_FPKM). **d** Top 10 biological functions of upregulated genes after R-spondin2 treatment using GO analysis. **e** GSEA comparison showing upregulation of mesoderm-associated genes with R-spondin2 treatment. **f** The kinetics of expression of representative mesoderm genes BRACHYURY, MIXL1, APLNR, and KDR during early hematopoietic differentiation from hESCs in chemically defined condition with or without treatment of R-spondin2 (20 ng/mL) analyzed with real-time PCR. Relative expression is normalized to the level (= 1) of control at day 0. Results are shown as means ± SD (*n* = 3). Results are shown as means ± SD (*n* = 3). *P < 0.05, ***P < 0.001

The ability of R-spondin2 to stimulate differentiation at the early stage led us to speculate that R-spondin2 might enhance the generation of mesoderm cells. Indeed, we found that the proportion of APLNR^+^ cells, a subpopulation of lateral-plate mesodermal cells that can be eventually converted into CD31^+^CD34^+^ HEPs [26], was elevated after R-spondin2 treatment in both H1 and BC1 cells (Additional file 5: Figure S4A, Fig. 4a; control 28.30% ± 4% vs R-spondin2 53.93% ± 1.64% *P* < 0.01, Additional file 5: Figure S4B). Consistently, the subsequent production of CD31^+^CD34^+^ HEPs was also enhanced (Additional file 5: Figure S4C, Fig. 4b; control 1.35% ± 0.04% vs R-spondin2 4.27% ± 0.25%, *P* < 0.01, Additional file 5: Figure S4D). Similar results were also obtained for the chemically defined differentiation system, therefore ruling out culture system dependency (Additional file 5: Figure S4E-F). Thus, R-spondin2 enhances hematopoiesis by augmenting APLNR^+^ mesodermal cells.

To further confirm the role of R-spondin2 in mesoderm formation, we sorted TRA-1-85^+^ cells from R-spondin2-treated H1 cells at day 2 of mAGM-S3 co-culture differentiation and analyzed gene expression profiles by performing RNA-SEQ [26, 38]. In accordance with enhanced mesodermal differentiation, R-spondin2 treatment caused significant enrichment of mesoderm-associated genes (Fig. 4c). In contrast, no significant difference was observed with respect to genes associated with other germ layers (Fig. 4c). Several cellular responses associated with mesoderm development such as mesoderm morphogenesis and mesoderm formation were enriched, as revealed by gene ontology (GO) of the upregulated genes in cells upon R-spondin2 treatment (Fig. 4d). Gene set enrichment analysis (GSEA) assay further confirmed significant changes (*P* < 0.05) in mesoderm-associated gene sets (Fig. 4e). We also validated these results by utilizing real-time RT-PCR. Consistently, representative genes associated with mesoderm development, such as BRACHYURY, MIXL1, APLNR, and KDR, were markedly upregulated from day 1 of differentiation (Fig. 4f). Together, these results suggest that R-spondin2 promotes hematopoietic differentiation of hPSCs by augmenting APLNR^+^ mesoderm cells.

### Activation of TGF-β signaling by R-spondin2

**Fig. 5:**
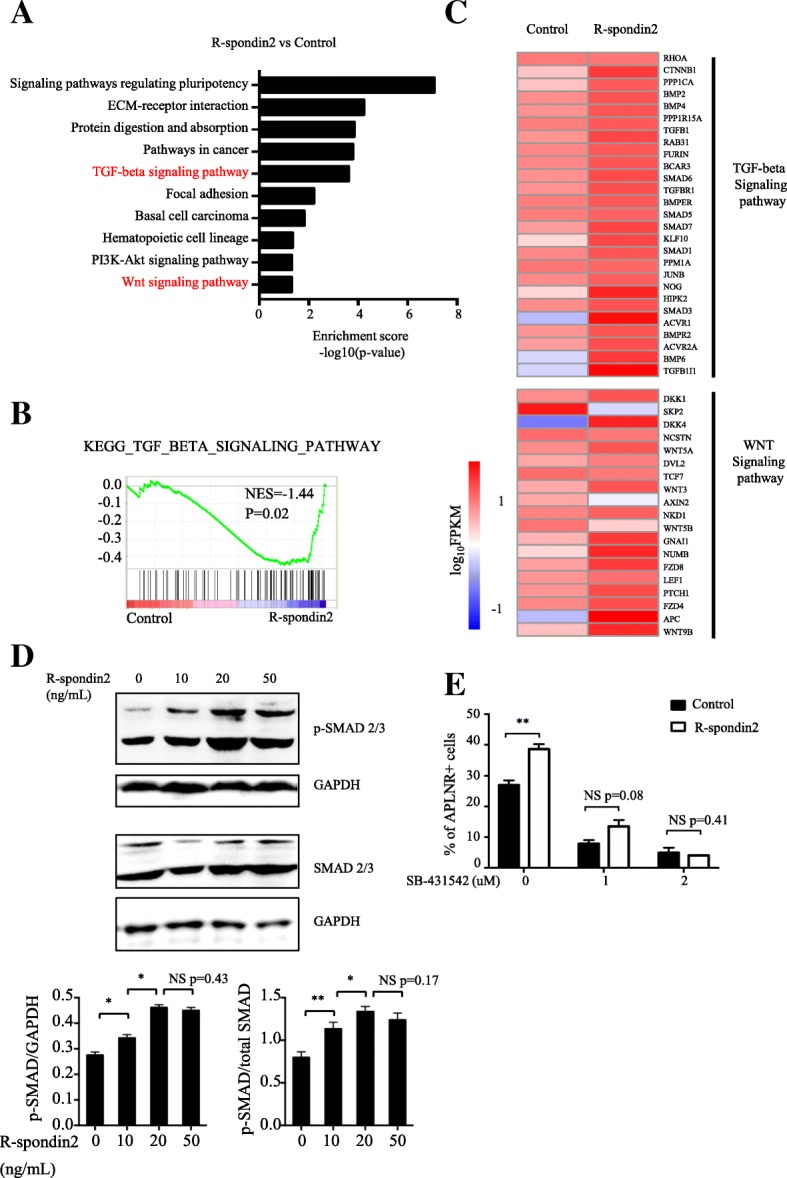
Activation of TGFβ signaling by R-spondin2. **a** KEGG pathway analysis of upregulated pathways in R-spondin2-treated cells versus control. **b** GSEA showing enrichment of TGFβ-associated genes with R-spondin2 treatment. **c** Heatmap showing genes with altered expression associated with TGFβ and WNT signaling after R-spondin2 treatment (log_10_FPKM). **d** Upper panel: Western blot analysis of phosphorylated SMAD2/3 and total SMAD2/3 protein levels in H1 cells at day 2 of differentiation in chemically defined medium treated with different doses of R-spondin2. 0, 10, 20, or 50 ng/mL of R-spondin2 was added at day 0 of hematopoietic differentiation. Lower panel: Densitometric quantitation of Western blot results of phosphorylated SMAD2/3 expression relative to GAPDH and total SMAD2/3 respectively. Results are shown as means ± SD (*n* = 3). NS, not significant. *P < 0.05. **P < 0.01. (E) Flow cytometry analysis of the percentage of APLNR^+^ cells from control and R-spondin2-treated H1 cells at day 3 of mAGM-S3 co-culture with different doses of SB-431542. R-spondin2 (20 ng/mL) was added along with 0, 1, or 2uM SB-431542 at day 0 of hematopoietic differentiation. Results are shown as means ± SD (*n* = 3). NS, not significant, **P < 0.01

To identify the downstream signaling pathways controlled by R-spondin2, we analyzed the RNA-SEQ results from cells with R-spondin2 treatment at day 2 of differentiation. KEGG analysis showed the enrichment of the WNT signaling pathway after R-spondin2 treatment, consistent with previous findings [33, 39] (Fig. 5a). Interestingly, among the significantly altered signaling pathways, enrichment of TGF-β signaling was even more significant than that of WNT signaling (Fig. 5a). GSEA confirmed that the enrichment of TGF-β signaling-associated gene sets in R-spondin2-treated cells was statistically significant (*P* = 0.02) (Fig. 5b). Consistently, as shown in the heatmap, a large number of genes associated with TGF-β signaling were upregulated after R-spondin2 treatment, including BMPs, TGFB1, ACVR1, and SMADs (Fig. 5c).

Because R-spondins have been widely shown as WNT agonists, here we mainly focused on investigating the function of TGF-β signaling. We found that R-spondin2 treatment caused a dose-dependent elevation of phosphorylated SMAD2/3, which is widely used as an indicator of TGF-β signaling activity (Fig. 5d). These results are in agreement with the bioinformatics analyses. To further determine whether activation of TGF-β signaling has functional significance, we tested whether SB-431542, an inhibitor of TGF-β signaling pathway, could block the enhancement of mesoderm development triggered by R-spondin2. Indeed, addition of SB-431542 inhibited the elevated generation of APLNR^+^ cells caused by R-spondin2 in a dose-dependent manner (Fig. 5e). When its dose reached 2 μM, SB-431542 nearly completely abolished the increase of APLNR^+^ cells induced by R-spondin2 treatment (Fig. 5e). Together, both the bioinformatics analyses and functional studies demonstrated that the effect of R-spondin2 on hematopoietic differentiation of hPSCs is mediated, at least partially, by the activation of TGF-β signaling.

### R-spondin2 ultimately augments the production of functional platelets

**Fig. 6:**
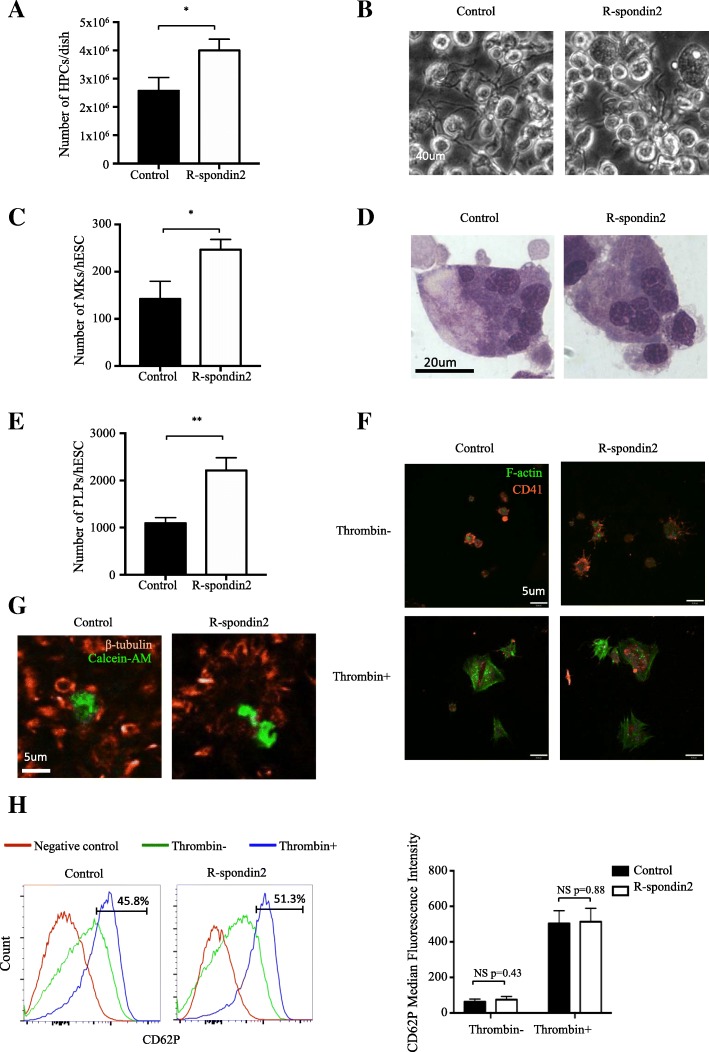
R-spondin2 ultimately augments the production of functional platelets from hPSCs. **a** Comparative analysis of the number of cobblestone-like HPCs after mechanical detachment from individual culture dishes of mAGM-S3 co-culture with or without R-spondin2 (20 ng/mL) treatment at day 1. Results are shown as means ± SD (*n* = 3). *P < 0.05. **b** Representative morphologies of large cells and proplatelets at day 6 of MK culture. **c** Quantitative analysis of CD41a^+^CD42b^+^ MKs generated from each H1 cells. Results are shown as means ± SD (*n* = 3). *P < 0.05. **d** Representative morphologies of multinuclear MKs by MGG (May-Grunwald-Giemsa) Staining at day 3 of megakaryocytic differentiation (scale bar, 20 mm). **e** Quantitative analysis of CD41a^+^CD42b^+^ PLPs generated from each H1 cells. Results are shown as means ± SD (*n* = 3). **P < 0.01. **f** Representative immunofluorescence images of hPSC-derived PLPs bound to immobilized fibrinogen with F-actin and CD41 stained in the absence (top) or the presence (bottom) of 1 U/mL thrombin. Scale bar = 5 μm. **g** Representative immunofluorescence images showing aggregation in a mixture of 2 × 10^5^ Calcein-AM (red)-labeled hPSC-derived PLPs and 2 × 10^7^ blood platelets. Scale bar = 5 μm. **h** Left panel: representative flow cytometry analysis of P-selectin (CD62P) in gated CD41a^+^ PLPs. Right panel: median fluorescence intensity analysis of CD62P in gated CD41a^+^ PLPs. Results are shown as means ± SD (*n* = 3). NS, not significant

We recently reported a three-step strategy for robust derivation of functional PLPs from hPSCs [26]. To test whether the HPCs generated with R-spondin2 treatment ultimately increased the final production of PLPs from hPSCs, we harvested the cobblestone-like HPCs from day 12 of hematopoietic differentiation with or without R-spondin2 treatment and induced them to form megakaryocytes (MKs) and gradually PLPs. Consistent with previous observations, upon R-spondin2 treatment, the co-culture procedure yielded a significant larger number of cobblestone-like HPCs per dish (Fig. 6a). When induced to undergo further MK differentiation, the HPCs from both the control and R-spondin2-treated cells are capable of generating large MK cells at day 6 of differentiation (Fig. 6b). Quantitative analysis of CD41a^+^CD42b^+^ MKs showed that R-spondin2 indeed increased the production of MK cells from H1 cells (Fig. 6c), while the MKs from both cultures showed high degree of polyploidy (Fig. 6d). Quantification of PLPs with flow cytometry analysis also showed increased PLP generation from H1 cells after R-spondin2 treatment (shown as per H1 cell; Fig. 6e). Furthermore, we measured the in vitro function of PLPs derived from this culture condition. We found that the derived PLPs from cells with or without R-spondin2 treatment were capable of adhering to immobilized fibrinogen in the presence of thrombin, indicating normal function of adhesion and spreading (Fig. 6f). In addition, the PLPs derived from R-spondin2-treated cells could aggregate upon thrombin stimulation (Fig. 6g). Analysis of P-selectin (CD62P) expression revealed little difference in the function of α-granule release for PLPs derived from cells with or without R-spondin2 treatment (Fig. 6h). Thus, enhanced HPC generation by early stimulation of R-spondin2 led to an ultimate increase in the final production of functionally intact PLPs. As such, this approach might be used as a powerful tool for in vitro large-scale generation of PLPs for future transfusion purposes.

## Discussion

In this study, we found that R-spondin2 can be produced by hematopoietic differentiation-supporting stromal cells and is capable of strongly promoting hematopoietic differentiation of hPSCs independently of culture conditions and cell lines. A short-term treatment during the early window of mesoderm induction with R-spondin2 enhances the generation of a subpopulation of APLNR^+^ cells more potent for hematopoietic differentiation, thus increasing the efficiency of HPC derivation. HPCs generated with R-spondin2 treatment are fully potent in further production of functional intact PLPs. Because of its potent effects on hematopoietic differentiation, we propose that R-spondin2 might potentially be used as a powerful tool for derivation of transplantable functional PLPs for translational medicine in the future.

R-spondin2 has been shown to play a pivotal role in embryogenesis. Rspo2-deficient mice display distal limb loss and lung hypoplasia in embryos and die immediately after birth due to respiratory failure [33, 40]. Nevertheless, the role of R-spondin2 in hematopoiesis has not been reported to date. While searching potential extracellular regulators of hPSC hematopoietic differentiation, we found from previously published sequencing results that R-spondin2 is highly expressed in active stromal cells, while this expression is validated [24, 32]. Interestingly, in this study we found that exogenous R-spondin2 can robustly promote hematopoietic differentiation and that this effect is independent of cell lines and culture conditions. Functionally intact PLPs can be further generated using the HPCs derived with R-spondin2 treatment. Therefore, our study identified, for the first time, the role of R-spondin2 in hematopoietic differentiation of hPSCs. Thus, R-spondin2 can be potentially applied as a powerful tool for large-scale generation of functional HPCs and mature blood cells, including platelets, for regeneration medicine.

hPSC hematopoietic differentiation goes through the process of mesoderm induction, HE generation, and EHT, mimicking the embryonic hematopoietic development in vivo [10]. We recently identified the role of MEIS1 in HE generation and MEIS2 in EHT, respectively [26, 27]. In this study, we further improved the understanding of the regulatory process by identifying a novel factor, R-spondin2, which confers its effect during the early window of mesoderm induction. As the origin of hematopoietic lineage, the mesoderm development in vivo and in vitro is under the regulation of several key signaling pathways [5, 8]. Among them, WNT signaling is one of the most critical factors as described previously [41]. For example, Wnt3 deficiency causes gastrulation defects and loss of primitive streak in mouse embryos [42]. During hPSC differentiation, activation of WNT signaling promotes mesoderm specification and consequently its derivative lineages, while WNT inhibition blocks this process [14]. Consistent with these observations, we found that a well-established WNT agonist, R-spondin2, is sufficient to significantly promote hematopoietic differentiation and does so by augmenting APLNR^+^ mesoderm subsets at the early stage of mesoderm induction. However, how R-spondin2 functions to stimulate this early population remains elusive. Therefore, it will be of great interest to explore the molecule mechanism underlying R-spondin2 function in mesoderm induction in future studies.

Interestingly, in addition to WNT signaling, we found that R-spondin2 can also activate TGFβ signaling to further regulate mesoderm development. To our knowledge, ours is the first study to reveal the functional link between R-spondin2 and TGFβ signaling. TGFβ signaling has been implicated in mesoderm specification both in vitro and in vivo [41]. For example, Smad2, one of the key signaling mediators of TGFβ signaling, is essential for early embryonic development [43, 44]. hPSC mesoderm differentiation requires activation of TGFβ signaling while treatment with chemical inhibitors of TGFβ signaling severely impairs hPSC hematopoietic differentiation [18, 45]. Our study reveals a functional connection between R-spondin2 and TGFβ signaling, which contributes to mesoderm differentiation, providing a mechanistic explanation of how R-spondin2 exerts its effects on mesoderm induction beyond the activation of WNT signaling. However, it remains to be determined how R-spondin2 modulates TGFβ signaling in hPSC hematopoiesis. A comprehensive delineation of the detailed mechanisms underlying the crosstalk between R-spondin2 and TGFβ awaits future studies.

## Conclusions

In this study, we found that R-spondin2 generated by hematopoiesis-supporting stromal cells significantly enhances hematopoietic differentiation of hPSCs. Supply of R-spondin2 exogenously at the early stage of mesoderm differentiation elevates the generation of APLNR^+^ cells. Furthermore, treatment of R-spondin2 ultimately augments the production of functional platelets from hPSCs. Mechanistically, R-spondin2 promotes the hematopoietic differentiation of hPSCs mediated, at least partially, by activating the TGF beta signaling pathway. Together, our discoveries represent important advances in dissecting the fundamental role of R-spondin2 in hPSC early hematopoiesis and provide a potential new strategy for large-scale generation of functional hematopoietic progenitors and mature blood cells for translational medicine.

## Additional files

Additional file 1: Supplemental Information. **Table S1.** The source of primary antibodies used in this study. **Table S2.** The source of fluorochrome-conjugated antibodies used in flow cytometry. **Table S3.** The primers used for real-time PCR. (DOC 66 kb)

Additional file 2: **Figure S1.** R-spondin2 promotes the generation of hematopoietic progenitors from hESCs. (PPT 1834 kb)

Additional file 3: **Figure S2.** R-spondin2 enhances hematopoietic differentiation of hPSCs independently of culture conditions and cell lines. (PPT 1634 kb)

Additional file 4: **Figure S3.** R-spondin2 treatment during early mesoderm differentiation suffices to promote hPSC hematopoietic differentiation. (PPT 382 kb)

Additional file 5: **Figure S4.** R-spondin2 promotes hematopoietic differentiation by augmenting APLNR^+^ mesodermal cells. (PPT 415 kb)
