# Supplementary material for: R-spondin2 promotes hematopoietic differentiation of human pluripotent stem cells by activating TGF beta signaling
Source: Stem Cell Res Ther. 2019 May 20;10:136. doi: 10.1186/s13287-019-1242-9 (PMC6528258; doi:10.1186/s13287-019-1242-9)
Supplement: Supplementary file 1 — Supplemental Information. Table S1. The source of primary antibodies used in this study. Table S2. The source of fluorochrome-conjugated antibodies used in flow cytometry. Table S3. The primers used for real-time PCR. (DOC 66 kb) [file 13287_2019_1242_MOESM1_ESM.doc]

**Supplemental Information**

**R-spondin2 promotes hematopoietic differentiation of human pluripotent stem cells by activating TGF-beta signaling**

Yv Wang^1,2^, Jie Gao^1,2^, Hongtao Wang^1,2^, Mengge Wang^1,2^, Yuqi Wen^1,2^, Jiaojiao Guo^3^, Pei Su^1,2^, Lihong Shi^1,2^, Wen Zhou^3*^, Jiaxi Zhou^1,2*^

**The file of Supplemental Figures and Text includes:**

Supplemental Figures (Figure S1- S4)

Supplemental Figure Legends

Supplemental Tables (Table S1- S3)

**Figure S1. R-spondin2 promotes the generation of hematopoietic progenitors from hESCs. Related to Figure 1**

(A) Representative photomicrographs of H1 cell colonies during differentiation in mAGM-S3 co-culture with (bottom) or without (top) the treatment of R-spondin2. Cobblestone-like hematopoietic cells are visible from day 7 as indicated by white arrows. Scale bar: 20μm, 100μm. (B) Representative flow cytometry analysis displaying the generation of CD43^+^ at day 7 of mAGM-S3 co-culture with or without the treatment of R-spondin2 (20ng/mL, with gating information). (C) Flow cytometry analysis of the percentage of CD43^+^ hematopoietic cells from H1 cells at day 7 in mAGM-S3 co-culture with the treatment of different doses of R-spondin2. 0, 10, 20, or 50 ng/mL of R-spondin2 was added at day 0 of hematopoietic differentiation. (D) Representative flow cytometry analysis displaying the generation of CD45^+^ at day 10 of mAGM-S3 co-culture with or without the treatment of R-spondin2 (20 ng/mL, with gating information). (E) Flow cytometry analysis of the percentage of CD45^+^ hematopoietic cells from H1 cells at day 10 in mAGM-S3 co-culture with the treatment of different doses of R-spondin2. 0, 10, 20, or 50 ng/mL of R-spondin2 was added at day 0 of hematopoietic differentiation. (F) Representative morphologies of BFU-E, CFU-E, CFU-GM, and CFU-GEMM from CFU culture of hESC-derived cells after 12 days of co-culture with (bottom) or without (top) the treatment of R-spondin2 (20ng/mL).

**Figure S2. R-spondin2 enhances hematopoietic differentiation of hPSCs independently of culture conditions and cell lines. Related to Figure 2**

(A) Representative immunofluorescence images of BC1 cells with or without the treatment of R-spondin2 (20ng/mL) showing the generation of CD45^+^ HPCs at day10 of mAGM-S3 co-culture. (B) Flow cytometry analysis of BC1 cells with or without the treatment of R-spondin2 (20ng/mL) showing the generation of CD45^+^ HPCs at day10 of mAGM-S3 co-culture. Results are shown as means ± SD (n = 3). *P<0.05. (C) Representative immunofluorescence images of H9 cells with or without treatment of R-spondin2 (20ng/mL) showing the generation of CD43^+^ HPCs at day 7 of chemically defined hematopoietic differentiation condition. (D) Flow cytometry analysis of H9 cells with or without treatment of R-spondin2 (20ng/mL) showing the generation of CD43^+^ HPCs at day 7 of chemically defined hematopoietic differentiation condition. Results are shown as means ± SD (n = 3). **P<0.01. (E) Representative immunofluorescence images of Z-15 cells with or without treatment of R-spondin2 (20ng/mL) showing the generation of CD43^+^ HPCs at day 7 of chemically defined hematopoietic differentiation condition. (F) Flow cytometry analysis of Z-15 cells with or without treatment of R-spondin2 (20ng/mL) showing the generation of CD43^+^ HPCs at day 7 of chemically defined hematopoietic differentiation condition. Results are shown as means ± SD (n = 3). **P<0.01. (G) Comparative analyses of number of CD43^+^ HPCs generated at day 7 of mAGM-S3 co-culture from a single seeded H1, BC1, H9, Z-15 cell with or without the treatment of R-spondin2 (20ng/mL). Results are shown as means ± SD (n = 3). *P<0.05, **P<0.01, ***P<0.001. (H) Comparative analyses of number of CD43^+^ HPCs generated at day 7 of chemically defined hematopoietic differentiation from a single seeded H1, BC1, H9, Z-15 cell with or without the treatment of R-spondin2 (20ng/mL). Results are shown as means ± SD (n = 3). *P<0.05, ***P<0.001.

**Figure S3. R-spondin2 treatment during early mesoderm differentiation suffices to promote hPSC hematopoietic differentiation. Related to Figure 3**

(A) Schematic diagram showing R-spondin2 treatment at different stages of differentiation and flow cytometry analysis for each population at indicated days of mAGM-S3 co-culture differentiation. (B) Flow cytometry analysis of the percentage of CD43^+^ HPCs at day 7 (left) and CD45^+^ HPCs at day10 (right) from BC1 cells in mAGM-S3 co-culture differentiation with R-spondin2 treatment (20ng/mL) at different stages of differentiation. (C) Schematic diagram showing R-spondin2 treatment at different window of mesoderm induction and flow cytometry analysis for each population in mAGM-S3 co-culture differentiation. (D) Flow cytometry analysis of the percentage of CD43^+^ HPCs at day 7 (left) and CD45^+^ HPCs at day 10 (right) from BC1 cells in mAGM-S3 co-culture differentiation with R-spondin2 treatment (20ng/mL) at different temporal window of mesoderm induction.

**Figure S4. R-spondin2 promotes hematopoietic differentiation by augmenting APLNR^+^ mesodermal cells. Related to Figure 4**

(A) Representative flow cytometry analysis with gating information displaying the generation of APLNR^+^ mesoderm cells at day3 of mAGM-S3 co-culture with or without the treatment of R-spondin2 (20ng/mL). (B) Flow cytometry analysis of the percentage of APLNR^+^ mesoderm cells at day 3 of differentiation in mAGM-S3 co-culture from BC1 cells with or without the treatment of R-spondin2 (20ng/mL). (C) Representative flow cytometry analysis with gating information displaying the generation of CD31^+^CD34^+^ at day5 of mAGM-S3 co-culture with or without the treatment of R-spondin2 (20ng/mL). (D) Flow cytometry analysis of the percentage of CD31^+^CD34^+^ HEPs at day 5 of differentiation in mAGM-S3 co-culture from BC1 cells with or without the treatment of R-spondin2 (20ng/mL). (E-F) Flow cytometry analysis of the percentage of APLNR^+^ mesoderm cells (E) at day 2 and CD31^+^CD34^+^ HEPs (F) at day 4 of differentiation in chemically defined system from H1 cells with or without the treatment of R-spondin2 (20ng/mL).

**Supplementary Table 1.**

The source of primary antibodies used in this study.

(I: immunofluorescence; W: western blotting)

| **Antibody** | **Source** | **Cat#** | **Dilution** |
| --- | --- | --- | --- |
| **CD43** | Santa Cruz | sc-51727 | 1:200(I) |
| **CD45** | Abcam | ab10558 | 1:200(I) |
| **GAPDH** | Affinity Biosciences | BF9210 | 1:10,000(W) |
| **Smad2/3** | Cell Signaling | 3102S | 1:1000(W) |
| **p-Smad2/3** | Cell Signaling | 8828S | 1:1000(W) |

**Supplementary Table 2.**

The source of fluorchrome conjugated antibodies used in flow cytometry.

| **Antigen** | **Fluorochrome conjugated** | **Clone** | **Source** | **Cat#** |
| --- | --- | --- | --- | --- |
| **TRA-1-85** | APC | Clone | R&D systems | FAB3195A |
| **hAPLNR** | APC | 72133 | R&D systems | FAB856A |
| **hCD31** | PE | WM29 | BD bioscience | 555446 |
| **hCD34** | APC | 8G12 | BD bioscience | 555824 |
| **hCD43** | APC | 1G10 | BD bioscience | 560198 |
| **hCD45** | APC | HI30 | BD bioscience | 555485 |
| **hCD41a** | APC | HIP8 | BD bioscience | 555751 |
| **hCD42b** | PE | HIP1 | BD bioscience | 555473 |
| **hCD62P** | PE | AK4 | BD bioscience | 555524 |

**Supplementary Table S3.**

The primers used for real-time PCR.

| **Gene** | **Forward Primer 5’-3’** | **Reverse Primer 5’-3’** |
| --- | --- | --- |
| ***ACTIN*** | CTCTTCCAGCCTTCCTTCCT | AGCACTGTGTGTTGGCGTACAG |
| ***RSPO1*** | TGGAGAGGAACGACATCCG | ACTTGGTGCAGAAGTTATGGC |
| ***RSPO2*** | CCTGCCCATCCGGGTACTAT | GCCTACTTTGCACTTGGTACAA |
| ***RSPO3*** | CTGTGTCCCCCAACAAATGAG | CAGTGCTGACTGATACCGATTTC |
| ***RSPO4*** | CCAGGAGGTCAACAGGTGC | CACTCCCGTGTGTTCTGGT |
| ***BRACHYURY*** | CTGGGTACTCCCAATGGGG | GGTTGGAGAATTGTTCCGATGA |
| ***MIXL1*** | TTTGGCTAGGCCGGAGATTAT | GGGCTTCAGACATTTCGTTTCAG |
| ***APLNR*** | CTCTGGACCGTGTTTCGGAG | GGTACGTGTAGGTAGCCCACA |
| ***KDR*** | GGCCCAATAATCAGAGTGGCA | TGTCATTTCCGATCACTTTTGGA |
| ***Actin*** | GACGGCCAGGTCATCACTATTG | AGGAAGGCTGGAAAAGAGCC |
| ***Rspo1*** | TGTGAAATGAGCGAGTGGTCC | TCTCCCAGATGCTCCAGTTCT |
| ***Rspo2*** | TTGCATAGAGGCCGCTGCTTT | CTGGTCAGAGGATCAGGAATG |
| ***Rspo3*** | GTACACTGTGAGGCCAGTGAA | ATGGCTAGAACACCTGTCCTG |
| ***Rspo4*** | CTCGCCCTGTACCGAAGGA | CACTTGCCGTACTGACGGATG |
