# Supplementary material for: R-spondin2 promotes hematopoietic differentiation of human pluripotent stem cells by activating TGF beta signaling
Source: Stem Cell Res Ther. 2019 May 20;10:136. doi: 10.1186/s13287-019-1242-9 (PMC6528258; doi:10.1186/s13287-019-1242-9)
Supplement: Supplementary file 2 — Figure S1. R-spondin2 promotes the generation of hematopoietic progenitors from hESCs. (PPT 1834 kb) [file 13287_2019_1242_MOESM2_ESM.ppt]

## Slide 1
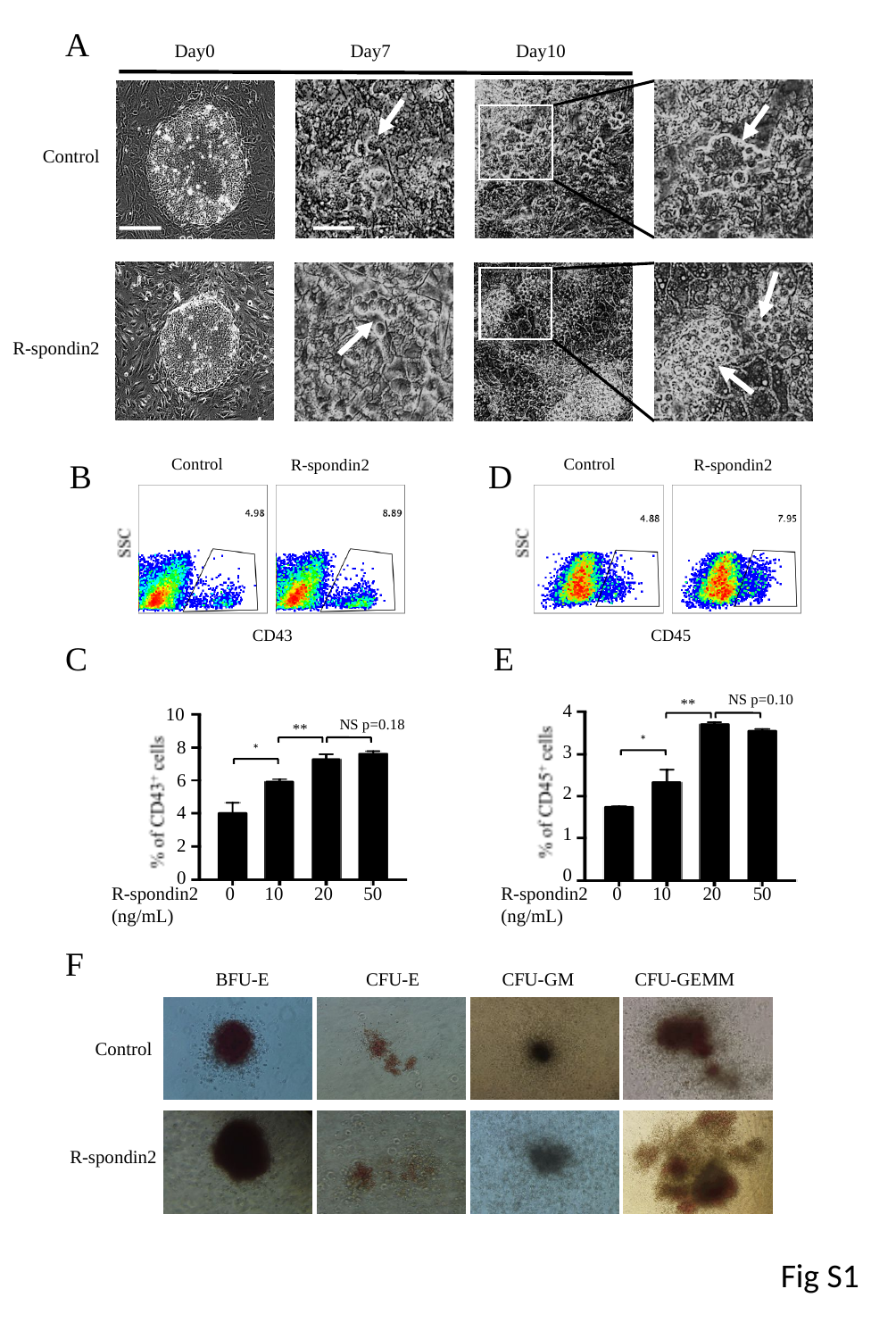

A
Day0
Day7
Day10
Control
20um
100um
R-spondin2
Control
Control
R-spondin2
R-spondin2
B
D
CD43
CD45
C
E
NS p=0.10
**
4
*
3
2
1
0
R-spondin2
(ng/mL)
0
10
20
50
10
NS p=0.18
**
8
*
6
4
2
0
R-spondin2
(ng/mL)
0
10
20
50
F
BFU-E
CFU-E
CFU-GM
CFU-GEMM
Control
R-spondin2
Fig S1
