# Supplementary material for: R-spondin2 promotes hematopoietic differentiation of human pluripotent stem cells by activating TGF beta signaling
Source: Stem Cell Res Ther. 2019 May 20;10:136. doi: 10.1186/s13287-019-1242-9 (PMC6528258; doi:10.1186/s13287-019-1242-9)
Supplement: Supplementary file 3 — Figure S2. R-spondin2 enhances hematopoietic differentiation of hPSCs independently of culture conditions and cell lines. (PPT 1634 kb) [file 13287_2019_1242_MOESM3_ESM.ppt]

## Slide 1
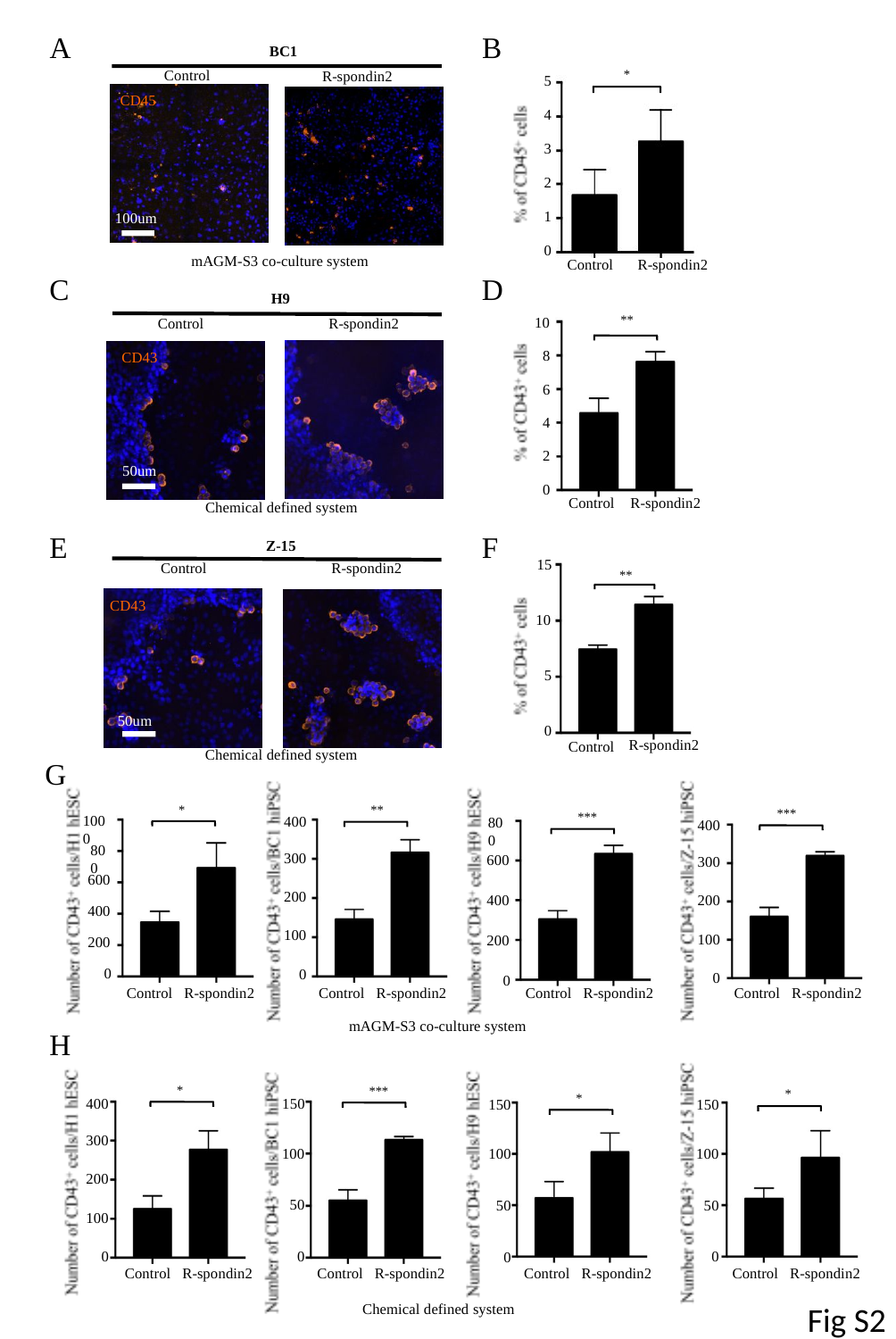

A
B
BC1
Control
*
R-spondin2
5
CD45
4
3
2
1
100um
0
mAGM-S3 co-culture system
Control
R-spondin2
C
D
H9
**
10
8
6
4
2
0
Control
R-spondin2
CD43
50um
Control
R-spondin2
Chemical defined system
E
F
Z-15
15
10
5
0
Control
R-spondin2
**
CD43
50um
R-spondin2
Control
Chemical defined system
G
*
**
***
***
1000
800
600
400
200
0
400
300
200
100
0
800
600
400
200
0
400
300
200
100
0
Control
R-spondin2
Control
R-spondin2
Control
R-spondin2
Control
R-spondin2
mAGM-S3 co-culture system
H
*
***
*
*
400
300
200
100
0
150
100
50
0
150
100
50
0
150
100
50
0
Control
R-spondin2
Control
R-spondin2
Control
R-spondin2
Control
R-spondin2
Chemical defined system
Fig S2
