# Supplementary material for: R-spondin2 promotes hematopoietic differentiation of human pluripotent stem cells by activating TGF beta signaling
Source: Stem Cell Res Ther. 2019 May 20;10:136. doi: 10.1186/s13287-019-1242-9 (PMC6528258; doi:10.1186/s13287-019-1242-9)
Supplement: Supplementary file 4 — Figure S3. R-spondin2 treatment during early mesoderm differentiation suffices to promote hPSC hematopoietic differentiation. (PPT 382 kb) [file 13287_2019_1242_MOESM4_ESM.ppt]

## Slide 1
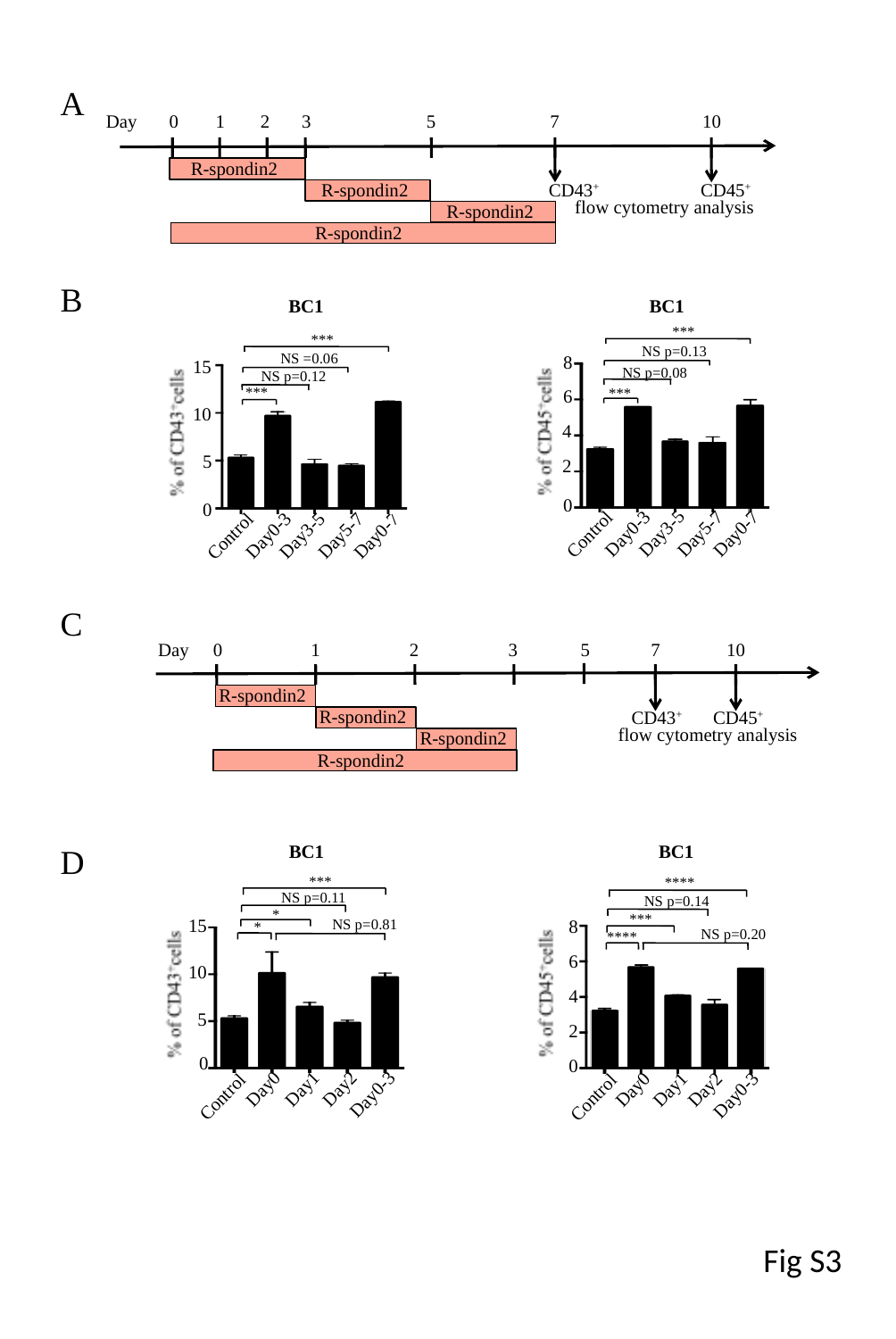

A
Day
0
1
2
3
5
7
10
R-spondin2
R-spondin2
CD43+
CD45+
flow cytometry analysis
R-spondin2
R-spondin2
B
BC1
BC1
***
***
NS p=0.13
NS =0.06
8
15
NS p=0.08
NS p=0.12
***
***
6
10
4
5
2
0
0
Day0-3
Day3-5
Day5-7
Day0-7
Control
Day3-5
Day0-3
Day5-7
Day0-7
Control
C
Day
0
1
2
3
5
7
10
R-spondin2
R-spondin2
CD43+
CD45+
flow cytometry analysis
R-spondin2
R-spondin2
BC1
BC1
D
***
NS p=0.11
*
15
NS p=0.81
*
10
5
0
Day0
Day1
Day2
Day0-3
Control
****
NS p=0.14
***
8
NS p=0.20
****
6
4
2
0
Day0
Day1
Day2
Day0-3
Control
Fig S3
