# Supplementary material for: R-spondin2 promotes hematopoietic differentiation of human pluripotent stem cells by activating TGF beta signaling
Source: Stem Cell Res Ther. 2019 May 20;10:136. doi: 10.1186/s13287-019-1242-9 (PMC6528258; doi:10.1186/s13287-019-1242-9)
Supplement: Supplementary file 5 — Figure S4. R-spondin2 promotes hematopoietic differentiation by augmenting APLNR+ mesodermal cells. (PPT 415 kb) [file 13287_2019_1242_MOESM5_ESM.ppt]

## Slide 1
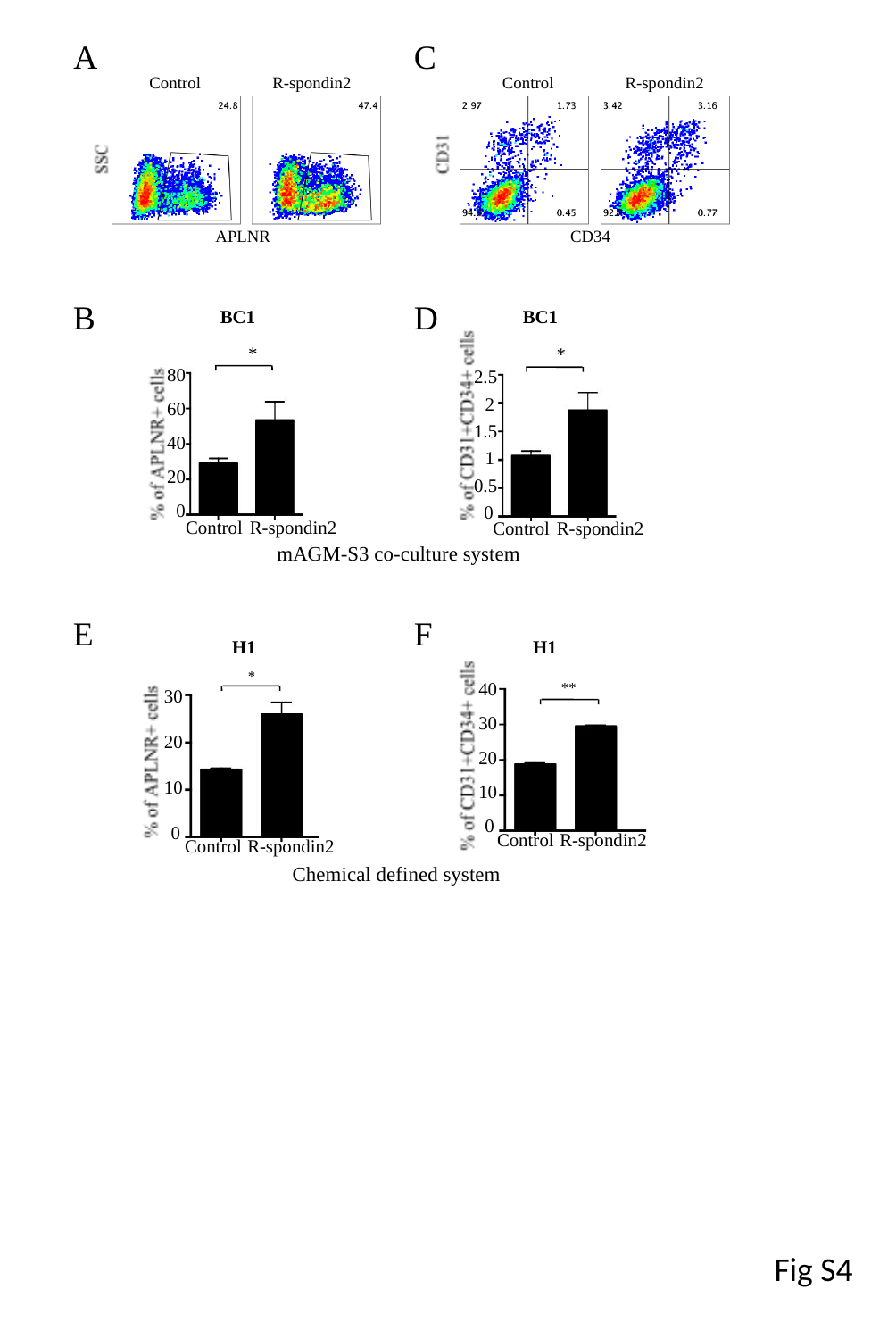

A
C
Control
R-spondin2
Control
R-spondin2
APLNR
CD34
B
D
BC1
BC1
*
2.5
2
1.5
1
0.5
0
Control
R-spondin2
*
80
60
40
20
0
Control
R-spondin2
mAGM-S3 co-culture system
E
F
H1
H1
40
30
20
10
0
**
Control
R-spondin2
*
30
20
10
0
Control
R-spondin2
Chemical defined system
Fig S4
